# Dynamics and risk sharing in groups of selfish individuals

**DOI:** 10.1016/j.jtbi.2023.111433

**Published:** 2023-04-07

**Authors:** Samuel Monter, Veit-Lorenz Heuthe, Emanuele Panizon, Clemens Bechinger

**Affiliations:** a University of Konstanz, Department of Physics, Universtaetsstrasse 10, Konstanz, 78464, Germany; b Centre for the Advanced Study of Collective Behaviour, Universtaetsstrasse 10, Konstanz, 78464, Germany; c The Abdus Salam International Centre for Theoretical Physics (ICTP), Strada Costiera 11 Trieste, 34151, Italy

**Keywords:** Collective behavior, Reinforcement learning, Prey predator interactions

## Abstract

Understanding why animals organize in collective states is a central question of current research in, e.g., biology, physics, and psychology. More than 50 years ago, W.D. Hamilton postulated that the formation of animal herds may simply result from the individual‘s selfish motivation to minimize their predation risk. The latter is quantified by the domain of danger (DOD) which is given by the Voronoi area around each individual. In fact, simulations show that individuals aiming to reduce their DODs form compact groups similar to what is observed in many living systems. However, despite the apparent simplicity of this problem, it is not clear what motional strategy is required to find an optimal solution. Here, we use the framework of Multi Agent Reinforcement Learning (MARL) which gives the unbiased and optimal strategy of individuals to solve the selfish herd problem. We demonstrate that the motivation of individuals to reduce their predation risk naturally leads to pronounced collective behaviors including the formation of cohesive swirls. We reveal a previously unexplored rather complex intra-group motion which eventually leads to a evenly shared predation risk amongst selfish individuals.

## Highlights

• Multi-agent reinforcement learning is applied to the “selfish herd” problem.
• Collective swirling motion of the entire group emerges.
• Supprisingly, the time average of predation risk is equal for all group members.

## 1 Introduction

Many animal species organize in groups, e.g., to gain benefits regarding foraging efficiency (Sumpter, 2010),   temperature  control (Szopek et al., 2013), energy considerations (Trenchard and Perc, 2016) or predation risk (Turner and Pitcher, 1986). The formation of such collective states is often described by social interaction rules which indeed leads to the formation of flocks, swirls and swarms as observed in many living systems (Galton, 1871, Couzin et al., 2003, Jens Krause, 2002, Parrish et al., 2002). As an alternative approach, W.D. Hamilton postulated the selfish herd hypothesis (SHH) which suggests that group formation of prey results from their selfish motivation to lower the predation risk (Hamilton, 1971). Within Hamilton’s simplified two-dimensional model, the predation risk of each individual is proportional to the area A of its Voronoi polygon (Boots et al., 1999), the latter being typically referred to as the domain of danger (DOD). When each individual attempts to minimize its DOD, this naturally leads to the formation of dense groups. Evidence supporting such geometric measure of predation risk is obtained from e.g., fiddler crabs (Viscido et al., 2002), seals (De Vos and O’Riain, 2010) and birds (Quinn and Cresswell, 2006). Although Hamiltons concept looks straightforward, the necessary motional strategies of individuals can be rather complex due to their competition for small DODs. In previous agent-based numerical simulations, the SHH has been studied by using simplifying motional rules aiming to achieve minimization of DODs (Hamilton, 1971, Viscido et al., 2002, Morton et al., 1994, James et al., 2004, Ose and Ohmann, 2017, Algar et al., 2019). However, such approaches do not warrant an optimal strategy of individuals. Furthermore, it typically leads to freezing of the group once agents have reached a minimal distance to neighbors. Therefore previous studies have mainly studied the transient process of group formation (Morrell et al., 2011) but did not investigate the complex intra-herd dynamics after aggregation.

In our study, we are using Reinforcement Learning (RL) to identify the unbiased and optimal strategy of selfish agents competing for their DODs. Compared to previous studies, this approach avoids the assumption of motional rules or social forces (alignment or cohesion) but provides an unbiased solution to the SHH. RL has already proven to be a useful tool for replicating natural behaviors, e.g., in the context of optimal response to predators and food sources (Durve et al., 2020, Young and Manh La, 2020, López-Incera et al., 2020). In our study we demonstrate the application of RL in the context of Hamiltons hypothesis. We observe the formation of a cohesive group which surprisingly displays a global rotation which has not been found in previous SHH-related simulations. In addition, we observe that agents permanently travel between the center and the edge of the group which eventually leads to an identical time-averaged DOD of all group members. This suggests that the selfish motivation of agents to reduce their DODs eventually leads to an equal predation risk of all group members.

## 2 MARL framework

In general, RL is a class of methods aiming to find the solution to an agent-based task in the form of a behavior, also called *policy*. The essence of most RL algorithms is an interaction loop between an agent and its environment: First, an observable – which is a representation of the current state – is given to the agent. Second, the agent uses the policy to choose an action as a response to its perception. Third, the system evolves in time due to the agent’s actions. Finally, a *reward* which is rating the new state is given to the agent. The series of states, actions and rewards is used to optimize the policy towards higher rewards. Notably, it is not only the instantaneous reward that is optimized, but the so-called *discounted return*, which also considers expected rewards in the future. The principles of RL can be extended to multi agent systems, termed Multi Agent Reinforcement Learning (MARL). In our study we apply the commonly used centralized training and decentralized execution (CTDE) paradigm (Foerster et al., 2016, Gupta et al., 2017, Zhang et al., 2021), where group members are represented by independent agents acting on individual observables and rewards, while they learn from shared experience (Gupta et al., 2017).

**Fig. 1:**
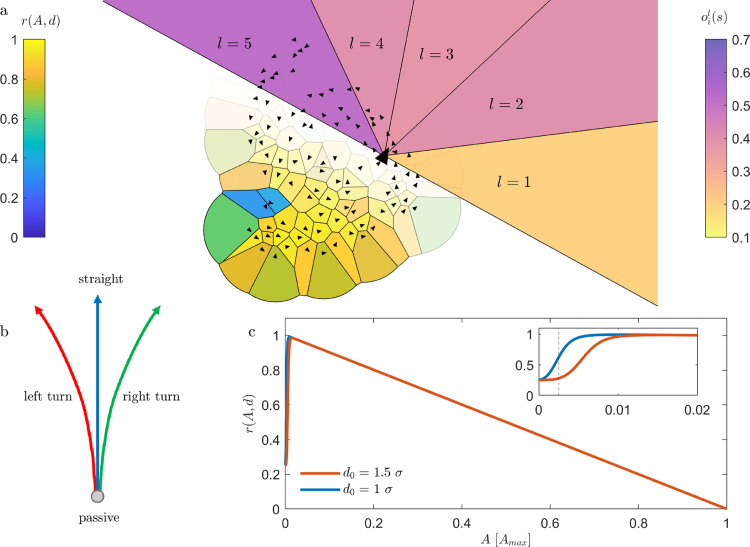
**a** Configuration of agents (black triangles) competing with each other regarding their Voronoi areas which are colored according to the corresponding reward r(A,d). Polygons at the group’s edge are limited by a cut off radius δ=10σ. Upper right: Representation of the vision cone of a single individual (highlighted by the larger triangle) which is divided into five sectors with 36°, each. The single sectors are colored according to the values $o_{i}^{l}\left(s\right)$ of the five dimensional observable $o_{i}\left(s\right)$. **b** Changes in position and orientation according to each of the 4 possible actions. Agents translate 0.25σ per non passive action and follow a trajectory with a constant radius of curvature 0.8σ during turning actions, while they change their orientation with a constant angular velocity $\omega =18\frac{\circ }{action}$. **c** Reward function for c=0.375 and $d_{0}=1\sigma$ and $d_{0}=1.5\sigma$, respectively. The vertical dashed line marks the onset of overlap. The curves are calculated under the assumption of a circular DOD around the rewarded agent with its next neighbor at a distance corresponding to the DOD’s diameter.

In our study we perform agent based simulations of groups of 100 individuals representing an animal herd. Each Individual is simulated as a disk with diameter σ. The state s at time t is quantified by an agent i by the observable $o_{i}\left(s\right)$ which represents a simple model of animal vision: each individual perceives its surrounding by a 180° vision cone which is divided in five equal sectors l=1−5 (Fig. 1a). Accordingly, $o_{i}\left(s\right)$ is a five-dimensional vector with each element $o_{i}^{l}\left(s\right)$ quantifying the crowdedness in the corresponding sector l. Each neighbor j contributes with $\frac{\sigma }{\left|r_{ij}\right|}$ to $o_{i}^{l}\left(s\right)$ with $\left|r_{ij}\right|$ the distance between i and j (Appendix A.6). Such description of visual perception where neighbors are weighted with their inverse distance is motivated by its established role in living systems (Viscido et al., 2002, Attanasi et al., 2014). In addition, we also consider sight-blocking effects which arise due to the finite diameter σ of individuals (Appendix A.6). As a result, the observable is a rather local quantity in particular at high densities. After each time step Δt and for each agent, the observable $o_{i}\left(s\right)$ is fed into a neural network (called *actor*), which produces the policy π(a|o). The policy gives the probabilities for the four possible actions a *move straight forward*, *turn left*, *turn right*, or *stand still* (Appendix A.5 and Fig. 1b), according to which the next action for each agent is chosen. During each action (except stand still) agents move by 0.25σ either straight or (in case of the turning actions) with a radius of curvature equal to 0.8σ. In addition, these actions are subjected to Gaussian white noise with a standard deviation of 20% (up to 50% the noise levels did not influence the qualitative outcomes). After the environment is updated using a simple MD simulation framework (Appendix A.5), a reward (as defined below) is given to each agent.

In the context of the SHH, the reward $r^{SHH}\left(A\right)$ must quantify the predation risk, the latter being proportional to the DOD. As reward we have chosen

(1) $$r^{SHH}\left(A\right)=\frac{A_{max}-A}{A_{max}-A_{min}}$$

where $A_{min}$ is the area $\pi {\left(\frac{\sigma }{2}\right)}^{2}$ of an individual. To avoid infinite values of the DOD A, a cutoff radius δ is usually applied (James et al., 2004), yielding a maximum possible area $A_{max}$. In the following we use δ=10σ, but qualitatively similar results are obtained for other values. With the above definition, $r^{SHH}$ varies between one and zero and decreases linearly with increasing DOD in accordance to the SHH. In addition, we add a second negative term to the reward which penalizes next neighbor distances d shorter than $d_{0}$. This proximity reward $r^{prox}\left(d\right)$ aims to mimic the attempt of collision avoidance as observed in many living systems. Couzin et al., 2002, Lukeman et al., 2010. We implemented $r^{prox}\left(d\right)$ in the form of

(2) $$r^{prox}\left(d\right)=-c\left[1-\tanh\left(\frac{d-d_{0}}{2}\right)\right]$$

**Fig. 2:**
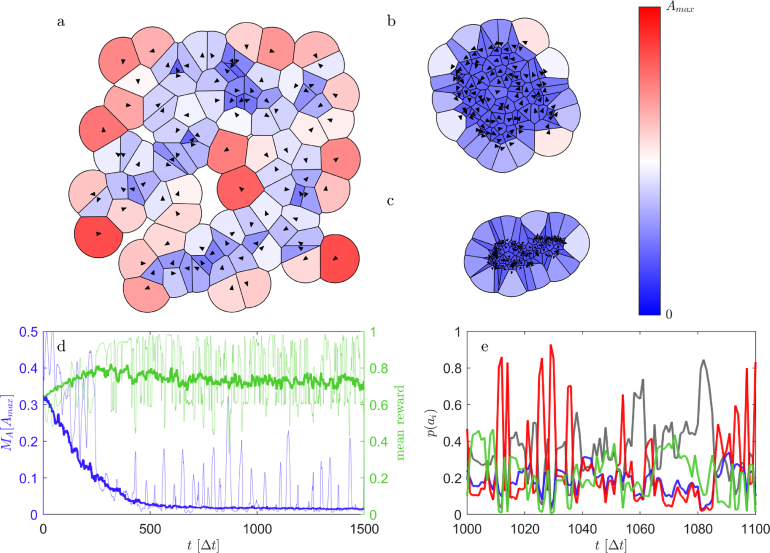
**a** Snapshots of agents (black triangles) in their starting random configuration and after aggregation. The examples shown in **b**, **c** correspond to the reward functions shown in Fig. 1c with parameters c=0.375 and $d_{0}=1.5\sigma$ and $d_{0}=1.0\sigma$, respectively. The Voronoi cells are colored according to their area. **d** Median of the area distribution (blue) and mean reward (green) of agents for c=0.375 and $d_{0}=1.5\sigma$ during the aggregation process. Thin lines show the corresponding data for a single, randomly selected agent. **e** Time series of the probabilities $p\left(a_{j}\right)$ in the NESS according to which a single agent chooses one of the four possible actions $a_{j}$ (actions color code identical to Fig. 1b).

The tanh is chosen to yield a step-like behavior with a smooth crossover to avoid discontinuities which are problematic in numerical simulations. The prefactor c and proximity threshold $d_{0}$ allow to adjust the onset and weight of $r^{prox}$, respectively. In what is discussed in the following we have set c=0.375 and $d_{0}=\sigma$ and $d_{0}=1.5\sigma$. A discussion how the choice of these parameters affects the resulting group behavior is provided in the SI. Fig. 1c shows the combined reward function $r=r^{SHH}+r^{prox}$ as a function of A for these parameters (since r depends both on A and d, we assume a circular DOD around the rewarded agent and its next neighbor at a distance corresponding to the DOD’s diameter in Fig. 1c).

To optimize (train) the actor network towards maximizing the returns we use the experience of all agents — in the form of observable, action and reward trajectories. The training is conducted in an episodic fashion with each training episode starting from a random configuration and orientation of agents (Fig. 2a) (for details see Appendix A.5). After typically 30 training episodes (equivalent to $30\times 3000\times 100=9\times 10^{6}$ individual actions), the MARL-algorithm has learned a policy π which describes how agents ideally respond to their observables in order to minimize their DOD. Using this policy, we can study the emergent dynamics of a selfish herd where each individual is driven solely by the motivation to reduce its predation risk according to the SHH.

## 3 Results

Fig. 2a–c show snapshots of a group of individuals in their initial random configuration and after having pursued their learned policy which maximizes their returns as defined above (for movies of the aggregation process and steady state we refer to the SI). The shown examples correspond to the reward functions shown in Fig. 1 c, for a full overview of the resulting group structures depending on the parameters c and $d_{0}$ we refer to the SI. As expected, during an episode the median of the DOD distribution $M_{A}$ continuously decreases over time until it converges to its final value (Fig. 2d). Notably, even though only small fluctuations are found in the median of the DOD $M_{A}$ and the mean reward, the DOD and the reward of individuals strongly vary in time (thin lines). This indicates a lively dynamics within the herd even after group formation is completed and the system has reached a non-equilibrium steady state (NESS). In Fig. 1e we show the time-dependent policy of one individual after the group has reached a NESS. Notably, even though the reward has already saturated, the probabilities for the different actions strongly fluctuate in time. This is due to the constant changes in the environment which causes permanent adjustments in the actions of agents and thus leads to a highly dynamic intra-group behavior in the NESS. In the following we will discuss this intra-group dynamics in more detail.

**Fig. 3:**
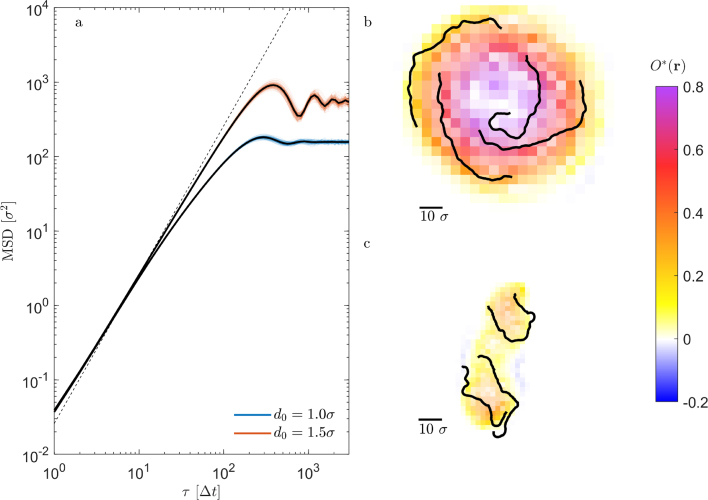
**a** MSD of individuals after having reached a steady state and for different parameters of the reward function c=0.375, $d_{0}=1.0\sigma$ (blue) and 1.5σ (red). Black lines show corresponding ensemble averages. Pronounced oscillations at intermediate time scales clearly indicate orbital motion. **b** Typical trajectories (black lines) with a duration of 230Δt for the reward parameters shown in blue in **a**. The colored background corresponds to the local rotational order parameter $O_{R}^{*}\left(r\right)$ which demonstrates a single vortex (being referred to in the following as strongly rotating group (SRG)). **c** same as **b** for the reward parameters of the red data in **a**. The color map shows a less pronounced global rotation and the formation of several local vortices (weakly rotating group (WRG)). The duration of trajectories corresponding to 150Δt.

### 3.1 Mean squared displacement

Fig. 3a shows the mean squared displacements (MSD) of individuals in their NESS for the two parameter sets of the reward used in our MARL simulations (Fig. 1c). The slight motion of the group’s center-of-mass has been subtracted for clarity. In both cases, we find an initial super diffusive behavior which eventually saturates above time intervals τ=1000Δt. In between, oscillations are visible which suggest a strong periodic orbital motional component, in agreement with the exemplary trajectories shown in Figs. 3b, c. Remarkably, the individual MSDs (thin lines) show only little deviations from the corresponding ensemble averages (thick lines). This suggests a pronounced collective motion, but also that the NESS are ergodic, i.e. each individual is able to traverse the entire group. This is confirmed by the trajectories which demonstrate the motion of individuals between the center and the edge of the group (Fig. 3b, c). Such behavior is not immediately obvious, since it is not clear why individuals at the group centers would move to the edges, thereby increasing their DOD.

**Fig. 4:**
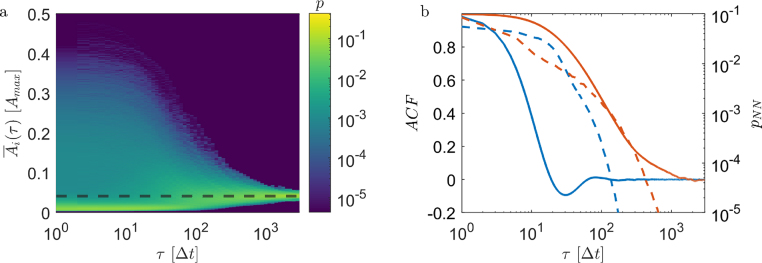
**a** Probability distribution of ${\overset{¯}{A}}_{i}\left(\tau \right)$ as a function of τ. Probabilities are shown in color and are normalized according to $\sum_{i}p\left({\overset{¯}{A}}_{i}\left(\tau \right)\right)=1$. The data converges to the ensemble average shown as a dashed line. **b** Ensemble averaged auto-correlation function (ACF) of the cumulative DOD (Eq. (5)) for a strongly and weakly rotating group (orange and blue solid lines, respectively). The dashed lines (same color code) show the probability $p_{NN}\left(\tau \right)$ that agents keep their next neighbors over time duration τ.

### 3.2 Rotational order

To characterize the collective motion of the group, we use the rotational order parameter (Bäuerle et al., 2020)

(3) $$O_{R}=\frac{1}{N}\overset{N}{\underset{i}{\sum }}\left|r_{i}\times u_{i}\right|$$

where the unit vectors $r_{i}$ and $u_{i}$ correspond to the position (relative to the group center) and orientation of individuals. For the reward parameters of Fig. 3b, we obtain $O_{R}\approx 0.6$ corresponding to a rather strong collective group rotation. This finding is supported by the spatially resolved rotational order parameter $O_{R}^{*}\left(r\right)$ (Appendix B) which displays a single vortex near the group center similar to what is frequently observed in living systems (Fig. 3b) (Jens Krause, 2002, Couzin et al., 2003, Camazine et al., 2003, Sumpter, 2010, Vicsek and Zafeiris, 2012). In the following we will refer to such collective states as a strongly rotating group (SRG). For the parameters corresponding to Fig. 3c, we find a much smaller value of the global rotation $O_{R}\approx 0.1$ and the formation of several local vortices which change their position over time (Fig. 3c). They will be referred to as weakly rotating groups (WRG).

### 3.3 Risk sharing

Despite a highly synchronized motion, individuals are in constant competition for their DODs even after the group formation process has been completed. This is seen when considering the cumulative averaged DOD in the NESS

(4) $${Ā}_{i}\left(\tau \right)=\frac{1}{\tau }\int_{0}^{\tau }A_{i}\left(t\right)dt$$

where τ is the averaging time interval (Fig. 4a). For small τ, ${\overset{¯}{A}}_{i}\left(\tau \right)$ varies between individuals but also strongly fluctuates for a given individual as a function of τ. This reflects the continuous attempt of individuals to reduce their DOD at the expense of their neighbors. Above τ≈500Δt, however, ${\overset{¯}{A}}_{i}\left(\tau \right)$ converges to the corresponding ensemble average, i.e. the average Voronoi areas eventually become almost identical for all individuals. In other words, despite their selfish motivation, on average the predation risk (DOD) becomes identical for all group members. To quantify the time after which the average DOD becomes approximately identical for all individuals, we also calculated the ensemble averaged auto-correlation function of the DOD in the NESS

(5) $$ACF\left(\tau \right)=\frac{1}{N}\overset{N}{\underset{i=1}{\sum }}\frac{\int_{0}^{T}\left(A_{i}\left(t\right)-{\overset{¯}{A}}_{i}\right)\left(A_{i}\left(t+\tau \right)-{\overset{¯}{A}}_{i}\right)dt}{\int_{0}^{T}{\left(A_{i}\left(t\right)-{\overset{¯}{A}}_{i}\right)}^{2}dt}$$

where ${\overset{¯}{A}}_{i}$ is the individual time averaged DOD. The solid lines in Fig. 4b which correspond to the examples shown in Fig. 3b, c yield correlation times of ∼100Δt and ∼1000Δt, respectively. These values are consistent with the corresponding rotation times of the groups. During this time, the neighborhood of initially adjacent peers is completely lost as shown by the corresponding probability distributions $p_{NN}\left(\tau \right)$ which measures the likelihood that initial neighbors remain neighbors after time τ (dashed lines in Fig. 4b).

## 4 Discussion

Using MARL we trained a group of selfish individuals to minimize their predation risk by reducing their DODs. In contrast to most previous SHH studies that relied on specific motional rules, in our approach we let the behavior emerge as an unbiased solution to the task. In agreement with Hamilton’s hypothesis (Hamilton, 1971), we found that individuals gather into cohesive groups. Notably, these groups exhibit a complex intra-herd dynamics leading to group rotation as observed in many living systems (Delcourt et al., 2016). Opposed to numerical simulations where rotating states are usually obtained by imposing alignment interactions between agents (Couzin et al., 2002, Vicsek et al., 1995), here such behavior results from the attempt of the agents to minimize their DODs. The appearance of swirls in context of the SHH can be understood by considering that edge positions of the group are less favorable (due to their larger DODs) compared to those near the group center. As a result, the motion of agents at the edges towards the groups center is rewarded. In addition, however, individuals aim to avoid collisions since close encounters are penalized. Keeping a balance between these two effects, eventually results in a preferred distance to neighbors which generally leads to group rotation (Delcourt et al., 2016, Vollmer et al., 2006, Nuzhin et al., 2021).

Most strikingly, we observe a fair risk distribution established amongst selfish individuals. This seems to be surprising because one would expect that agents near the group center would never abandon such positions having low (compared to edge positions) predation risk. However, agents do not have full control regarding their DODs because they also depend on the positions of their neighbors. As a result, agents must permanently respond to the motion of their peers which causes the system to remain fully ergodic. Therefore all agents will move through entire group which then leads to identical time-averaged DODs. We wish to emphasize that all behaviors reported in our study remain stable upon variations of parameters such as the vision angle, the strength of noise and even when visual blocking of neighbors is entirely removed. In all cases, an equal distribution of risk is established over time.

## 5 Conclusion

In summary, we have investigated the intra-herd dynamics which evolves within a group of individuals competing with each other to reduce their predation risk. Using a MARL framework, we demonstrated that group members first assemble in a cohesive group which exhibits pronounced collective properties. Depending on the specific reward parameters, groups with different topologies are observed, ranging from strongly to weakly rotating groups and rings. Notably, the selfish motivation of individuals to minimize their DOD eventually leads to a fair risk distribution amongst all group members. Our results therefore indicate that Hamilton’s hypothesis indeed provides an explanation to describe gregarious behavior in living systems.

## CRediT authorship contribution statement

**Samuel Monter:** Performed the numerical simulations, Analyzed the results, Wrote the computer code for the MD environment, Discussion of the results, Writing – original draft. **Veit-Lorenz Heuthe:** Devised the project, Performed the numerical simulations, Analyzed the results, Wrote the computer code for the MD environment, Discussion of the results, Writing – original draft. **Emanuele Panizon:** Wrote the computer code for the MARL algorithm, Discussion of the results, Writing – original draft. **Clemens Bechinger:** Devised the project, Discussion of the results, Writing – original draft.

## Declaration of Competing Interest

The authors declare that they have no known competing financial interests or personal relationships that could have appeared to influence the work reported in this paper.

## Figures and Tables

**Fig. A.5:**
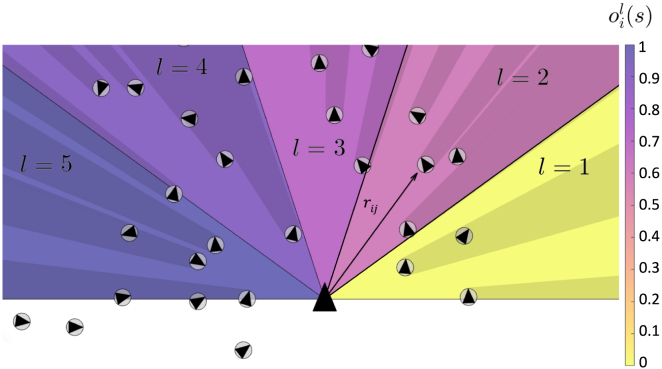
Agents are able to perceive their environment by a vision cone of 180° which is divided in five identical sectors l=1−5. Using the number of peers $M_{l}$ and distance $\left|r_{ij}\right|$ between individual i and its peers j in each sector, the value $o_{i}^{l}\left(s\right)$ is calculated. Shadows represent how perceived individuals block the perception of peers behind them, which is captured in the parameter $\phi_{ij}$.

## Appendix A MARL framework

### A.1 Single agent Reinforcement Learning with PPO

To describe the optimization process of the policies, we will first present the main ingredients for a single agent, and then how these are applied to the case of multiple agents. Model-free Reinforcement Learning is the field which studies the process of optimizing how to interact with an environment in response to previous experience. The experience is given by trajectories, which are sequential collections of states of the environment $s_{t}$, performed actions $a_{t}$ and obtained rewards $r_{t}$: $\tau =\left\{s_{0},a_{0},r_{1},s1,a_{1},r_{2}\ldots s_{T},a_{T},r_{T+1}\right\}$. From the trajectories, one can define the value function as the expected cumulative return of starting in a state and following the policy π, i.e

(A.1) $$V=E_{\tau ∼\pi }\left[\underset{t}{\sum }\gamma^{t}r_{t+1}|s_{0}=s\right].$$

The goal of learning is to optimize the total return, defined as $G=E_{\tau ∼\pi }\left[\sum_{t}\gamma^{t}r_{t+1}\right]$, changing the agent’s policy π. We use an on-policy actor–critic algorithm,   called   Proximal   Policy   Optimization (PPO) (Schulman et al., 2017), which uses two Artificial Neural Networks (ANNs) as function approximations for the value function $V_{\pi }\left(s\right)\approx V_{w}\left(s\right)$ and the policy $\pi_{\theta }\left(a,s\right)$. w and θ refer to the parameters of the actor and policy ANNs respectively. PPO is an algorithm which uses the policy gradient theorem, which – in its original “vanilla” form – shows that one can use experienced trajectories τ for evaluating the gradient of the return G with respect to the (parameters of the) the policy:

(A.2) $$\nabla_{\theta }G=E_{\tau ∼\pi }\left[\underset{t}{\sum }A_{\pi }\left(s_{t},a_{t},r_{t+1}\right)\nabla_{\theta }\log\pi_{\theta }\left(a_{t}|s_{t}\right)\right]$$

Here, $A_{\pi }\left(s_{t},a_{t},r_{t+1}\right)$ can be any of the advantage functions (Schulman et al., 2015) for the current policy, and in essence estimates how valuable the action $a_{t}$ is, given the observable $s_{t}$, and the experienced reward $r_{t+1}$. A common choice for the advantage function is the temporal difference error (also known as TD(0)) which is evaluated as

(A.3) $$A_{\pi }^{TD\left(0\right)}\left(s_{t},a_{t},r_{t+1}\right)=r_{t}+\gamma V_{\pi }\left(s_{t+1}\right)-V_{\pi }\left(s_{t}\right)$$

$V\left(s_{t}\right)$ here is the (unknown) value of state $s_{t}$. In actor–critic algorithms, the value function V appearing in the advantage function is approximated by an ANN, $V_{\pi }\left(s_{t}\right)\approx V_{w}\left(s_{t}\right)$, which is indeed the *critic* of the algorithm.

In this work we use the generalized advantage estimation (GAE) (Schulman et al., 2015), which has been shown to result in faster and more stable convergence than pure TD(0).

The standard update of the parameters θ is to follow the direction of the gradient with a small step parameterized by some learning-rate, i.e. performing steepest descent with the constraint that the distance in parameter space is sufficiently small. The difference between the PPO algorithm compared to the “vanilla” policy-gradient algorithm is in the update step of the actor. It was proposed (Kakade, 2001) that a better method should consider constraints based on the distance between subsequent policies and not the underlying parameters.

To solve this issue several proposals in RL have been made, such as Natural Policy Gradient (Kakade, 2001, Bhatnagar et al., 2009) and Trust Region Policy Optimization (Schulman et al., 2015), where the Kullback–Leibler distance between old and new policy is taken explicitly in consideration. However these methods can be rather complex and cumbersome from a computational point of view.

PPO, and specifically the PPO-Clip variant we implement (Schulman et al., 2017), solve the issue in an approximate (but computationally very efficient) way, effectively imposing a limit to the maximum amount of policy change so that the ratio of new and old policies cannot go beyond a fixed range [1−ϵ,1+ϵ]. Details of the GAE and PPO implementation in the numerical simulations are discussed in Appendix A.3.

### A.2 The MARL framework

For a comprehensive overview of MARL we refer to the literature (Zhang et al., 2021). Here, we only provide some general information being important for our study. The central aspect of the MARL framework is that the evolution of the environment, i.e. its dynamics, is determined by multiple agents each executing specific actions. In our study the environment consists of N members of a herd (each an individual agent) whose configuration is fully described by the (2+1)N positional and orientational degrees of freedom. Such configuration at time t is the state $s_{t}$ of the environment as described above.

In our case, however, individual agents do not have a complete, global view of the whole environment. Instead, they have to rely only on partial information, i.e. their individual observations $o_{i,t}=o_{i}\left(s_{t}\right)$. Actions are then taken from probability distributions, the policies π, i.e, $a_{i,t}∼\pi \left(a|o_{i,t}\right)$, . We employ the CTDE paradigm, where policies are shared by the agents - i.e. $\pi_{i}=\pi_{j}=\pi$. In our case, the policy function is implemented by a single ANN, parameterized by parameters θ, such that $\pi =\pi_{\theta }\left(a|o\right)$.

After all agents have (independently) selected their actions $\left\{a_{i,t}\right\}$ at time t, the system evolves into a new state $s_{t+1}$ which is computed via a molecular dynamics (MD) simulation. In the new state, each agent is assigned a reward $r_{i,t+1}=r_{i}\left(s_{t+1}\right)$.

These steps are repeated a finite number of times T for different episodes, each starting from a random configuration. For each episode, and for each agent, we obtain a set of individual trajectories $\tau_{i}=\left\{o_{i,0},a_{i,0},r_{i,1},oi,1,a_{i,1},r_{i,2}\ldots o_{i,T},a_{i,T},r_{i,T+1}\right\}$.

The purpose of MARL is for the agents to maximize their expected return $G_{i}$, defined as a decaying cumulative sum of rewards $G_{i}=E_{t∼\pi }\left[\sum_{t}\gamma^{t}r_{i,t+1}\right]$, where τ∼π means that the trajectories are sampled following the policy π and γ∈[0,1] is the so-called discount factor, which determines the importance in the present of future rewards (a reward coming m steps in the future is valued $\gamma^{m}$). Since all agents share the same policy and are initialized with the same distribution, their expected return is the same: $G_{i}=G$ for all i. Within the framework of CTDE, we use an approximate version of the policy-gradient theorem, i.e.:

(A.4) $$\nabla_{\theta }G\approx 1/N\underset{i}{\sum }E_{\tau_{i}∼\pi }\left[\underset{t}{\sum }A_{\pi }\left(o_{i,t},a_{i,t},r_{i,t+1}\right)\nabla_{\theta }\log\pi_{\theta }\left(a_{i,t}|o_{i,t}\right)\right].$$

This approximation neglects that changes in θ do not only affect directly the return of the agent i but also modify indirectly its environment, through the change in other agents’ behavior. Due to the non-stationarity of the environment, in general the learning process in MARL can become unstable and convergence is not always guaranteed. Notably, however, all results presented in our study show convergence of the policy and of the average rewards.

### A.3 MARL implementation

The general procedure for the MARL implementation is as follows. First, the ANNs parameters are initialized at random. Individual trajectories are collected and stored in memory: $\tau_{i}=\left\{o_{i,0},a_{i,0},r_{i,1},o_{i,1},a_{i,1},r_{i,2}\ldots o_{i,T},a_{i,T},r_{i,T+1}\right\}$. From the trajectories, the TD errors and empirical returns are calculated for each agent and each time, respectively

(A.5) $$\delta_{i,t}=r_{i,t}+\gamma V_{w}\left(o_{i,t+1}\right)-V_{w}\left(o_{i,t}\right)$$

and,

(A.6) $$R_{i,t}=\overset{T-t-1}{\underset{k=0}{\sum }}\gamma^{k}r_{i,t+1}+\gamma^{T-k}V_{w}\left(o_{i,T}\right)$$

The advantage function is calculated from the $\delta_{i,t}$:

(A.7) $$A_{i,t}^{GAE}=\overset{T-t}{\underset{k}{\sum }}{\left(\lambda \gamma \right)}^{k}\delta_{i,t}^{k}.$$

To update the parameters of the ANNs, two objective functions have to be obtained. First, the PPO objective is calculated as the average over all agents from the modified policy-gradient expression of (A.4):

(A.8) $$L_{PPO}\left(\theta^{\prime }\right)=1/N\underset{i,t}{\sum }\min\left(\frac{\pi_{\theta }\left(a_{i,t}|o_{i,t}\right)}{\pi_{\theta^{\prime }}\left(a_{i,t}|o_{i,t}\right)}\overset{GAE}{\underset{i,t}{A}},g\left(\epsilon ,A_{i,t}^{GAE}\right)\right)$$

Where g(ϵ,A)=(1+ϵsign(A))A. The policy parameters θ are then chosen to minimize $L_{PPO}\left(\theta^{\prime }\right)$. We use a gradient descent method, using the standard back-propagation method for numerical differentiation of ANNs, as implemented in the tensorflow platform (Abadi et al., 2015). The value function is updated to minimize the mean squared error between the evaluated values $V_{w}$ and the empirical returns

(A.9) $$L_{V}=\frac{1}{N}\underset{i,t}{\sum }{\left(R_{i,t}-V_{w}\left(o_{i,t}\right)\right)}^{2}$$

After the parameters have been changed, new trajectories are computed with the updated policy, and the cycle is repeated.

### A.4 Artificial neural networks

For both the actor and critic ANN we use an input layer with 5 nodes corresponding to the observable vector $o_{i}\in R^{5}$. Three hidden layers with 32, 16 and 16 nodes respectively follow. The layers are densely connected and we use the ReLu non linear activation function. For the critic ANN no activation function is used for the output layer, which is just a scalar. For the actor ANN the final layer is of size 4 equaling the number of actions and softmax activation is applied so that it is indeed a probability distribution according to which actions can be chosen. Both ANNs are initialized with random weights.

### A.5 Numerical simulations

Simulations for training and evaluation episodes are run with simple overdamped Langevin dynamics. A full training episode has 3000 time steps Δt, each time step corresponds to one observable-action-reward cycle. For training the ANNs 100 episodes are run. The parameters for the discount and the GAE are set to λ=0.97 and γ=0.95 respectively for all training runs. At the beginning of each episode, 100 individuals are randomly distributed on a square lattice with a spacing of two agent diameters within an area of 100σ×100σ. The direction of orientation is chosen at random.

For each agent i, an observable $o_{i,t}$ is calculated from the current state. Each agent chooses an action $a_{i,t}$ according to the current policy $\pi =\pi_{\theta }\left(a|o_{i,t}\right)$ from the 4 options; move straight, turn left/right or stand still. Then, the system is propagated using a simple Euler scheme MD approach with N = 60 integration steps during the execution of one action.

In the case of the move straight action agents move with a constant velocity v=0.25σ action in the direction of their current orientation. During the turning actions individuals move on a curved trajectory with a radius of curvature equal to 0.8σ with the same velocity. During this the orientation is changed with a constant angular velocity of $\omega =18\frac{\circ }{action}$. While being in the passive action individuals do not alter their orientation or position by their own intent. The translational and angular velocities of individuals are subject to Gaussian noise, with a standard deviation of 0.2v or 0.2ω, respectively.

To avoid agent overlap, we added a short-ranged repulsion interaction ($U\approx r_{ij}^{12}-r_{ij}^{6}$) with a cutoff at r=σ. After execution of an action, new observables $o_{i,t+1}$ and rewards $r_{i,t+1}$ are calculated for each agent. Above steps are repeated 3000 times during one training episode. During each episode, actor and critic ANN are updated every T=128 actions with the experience of all agents, collected in the form of trajectories $\tau_{i}=\left\{o_{i,0},a_{i,0},r_{i,1},o_{i,1},a_{i,1},r_{i,2}\ldots o_{i,T},a_{i,T},r_{i,T+1}\right\}$. A total of 100 subsequent training episodes are run for the complete training of one set of actor and critic ANNs.

To investigate the emergent group behavior, evaluation simulations are run in the same way as explained above, just without training the ANNs. The length of these simulations has been adjusted to up to 100000 actions, depending on the requirements of the data analysis.

### A.6 Observable definition

The observable vector $o_{i}\left(s\right)$ is a simplified representation of animal vision. As a first simplification step, the surrounding is divided into 5 equal sectors spanning in total a vision cone of 180° at the front of the central individual i. For each sector a scalar $o_{i}^{l}\left(s\right)$ is calculated. Each of these scalars represents the crowdedness in each sector. Every perceived individual j contributes with $\phi_{ij}\frac{\sigma }{\left|r_{ij}\right|}$, where σ is the individual diameter and $\left|r_{ij}\right|$ the distance between i and j. The weighting with the inverse distance to neighbors has been argued to represent a realistic description of animal vision (Viscido et al., 2002). $\phi_{ij}$ scales between 0 and 1 to account for the fraction of the silhouette of individual j that is blocked by other individual in between individuals i and j (as visualized by the shadows in Fig. A.5). The value of the contribution of each perceived individual j is limited to 1 using a min function to prevent diverging values for short distances. Summing up all contributions of $M_{l}$ individuals in the sector with index l yields:

(A.10) $$o_{i}^{l}\left(s\right)=\overset{M_{l}}{\underset{j\neq i}{\sum }}min\left(\phi_{ij}\frac{\sigma }{\left|r_{ij}\right|},1\right)$$

Generally $o_{i}^{l}\left(s\right)$ can be thought of as a measure for the coverage of an individual’s horizon within one sector. The complete observable is a vector $o_{i}\left(s\right)$ containing the five values $o_{i}^{l}\left(s\right)$.

## Appendix B Local rotational order parameter

The local rotational order parameter $O^{*}\left(r\right)$ gives a spatially resolved measure for the vorticity of a group of polar entities. It is derived from the rotational order parameter $O_{R,i}$ which is defined as the magnitude of the cross product between the unit vector pointing from the chosen center of rotation r to the position of the individual $p_{i}$ and the unit vector derived from the individual orientation $\theta_{i}$. To achieve spatial resolution over r the $O_{R,i}$ for all individuals are summed up and weighed with a Gaussian kernel according to their distance to the center of rotation $\left|r_{i}\right|$. The variance $\sigma^{2}$ is scaled to achieve maximal visibility of local vortices and cancel out edge effects. Since the system of interest is two dimensional, vectors are extended to the 3rd dimension by appending a zero in order to use the cross product.

(B.1) $$O^{*}\left(r\right)=\frac{1}{N}\overset{N}{\underset{i=1}{\sum }}\frac{O_{R,i}\exp\left(\frac{-{\left|r_{i}\right|}^{2}}{2\sigma^{2}}\right)}{\exp\left(\frac{-{\left|r_{i}\right|}^{2}}{2\sigma^{2}}\right)}$$

$$\text{with}\;O_{R,i}=\left|r_{i}\times u_{i}\right|$$

$$r_{i}=\frac{p_{i}-r}{\left|p_{i}-r\right|}$$

$$u_{i}=\left(cos\left(\theta_{i}\right),sin\left(\theta_{i}\right),0\right)$$

## Appendix C Supplementary data

Video S1 Aggregation of the strongly rotating group (SRG)

Video S2 on equilibrium steady state (NESS) of the strongly rotating group.

Video S3 on equilibrium steady state (NESS) of the weakly rotating group.

MMC S4 Information about parameter combinations not discussed in the main text and video descriptions.

MMC S5 .

MMC S6 .

MMC S7 .
