# Supplementary material for: Dynamics and risk sharing in groups of selfish individuals
Source: J Theor Biol. 2023 Apr 7;562:111433. doi: 10.1016/j.jtbi.2023.111433 (PMC10020420; doi:10.1016/j.jtbi.2023.111433)
Supplement: MMC S4 — Information about parameter combinations not discussed in the main text and video descriptions. [file mmc4.pdf]

Supplementary Information:
Dynamics and Risk Sharing in Groups of Selfish Individuals

Veit-Lorenz Heuthe[a,b], Samuel Monter[a], Emanuele Panizon[c], Clemens Bechinger[a,b]

[a] *University of Konstanz, Department of Physics, Universtätstraße 10, Konstanz, 78464, Germany*
[b] *Centre for the Advanced Study of Collective Behaviour, Universtätstraße 10, Konstanz, 78464, Germany*
[c] *The Abdus Salam International Centre for Theoretical Physics (ICTP), Strada Costiera 11 Trieste, 34151, Italy*

---

1. Phase Diagram

The d_0 vs. c parameter space is scanned with multiple training runs. The RL algorithm converges towards different group behaviors depending on the parameter set. The observed behaviors are sorted into categories qualitatively which yields the phase diagram shown in Fig. 1. Snapshots of the characteristic group geometries are shown next to it. The characteristics of the phases can be shortly described as follows:

> **Compact Cluster:** Individuals aggregate quickly into multiple dense clusters, which then further coalesce. The clusters are dynamic in shape, but do not move in a coordinated fashion.
>
> **Collective Group:** Individuals reduce their distance and start to perform a collective rotation. This class can be subdivided into strongly rotating groups and weakly rotating groups.
>
> **Ring:** Individuals form a ring which rotates around its center of mass, this is however not stable in the long term. Initially formed geometries will either curl up into multiple smaller subgroups for intermediate c and d_0 or fragment for high c and d_0. Since this is not the behavior of interest it will not be discussed further.

The order in the phase diagram can be decomposed into two trends following the two varied parameters. At low c, clusters are formed since collisions are not sufficiently punished. In this region of low c the proximity threshold d_0 does not have a significant influence. Above a threshold of c more complex behaviors emerge. The kind of behavior is determined by the proximity threshold d_0. With increasing d_0, the radii of orbital motion of individuals grow larger with group structures changing from geometries consisting of multiple sub-swirls to one collectively rotating swirl. For intermediate d_0 swarm-like geometries form.

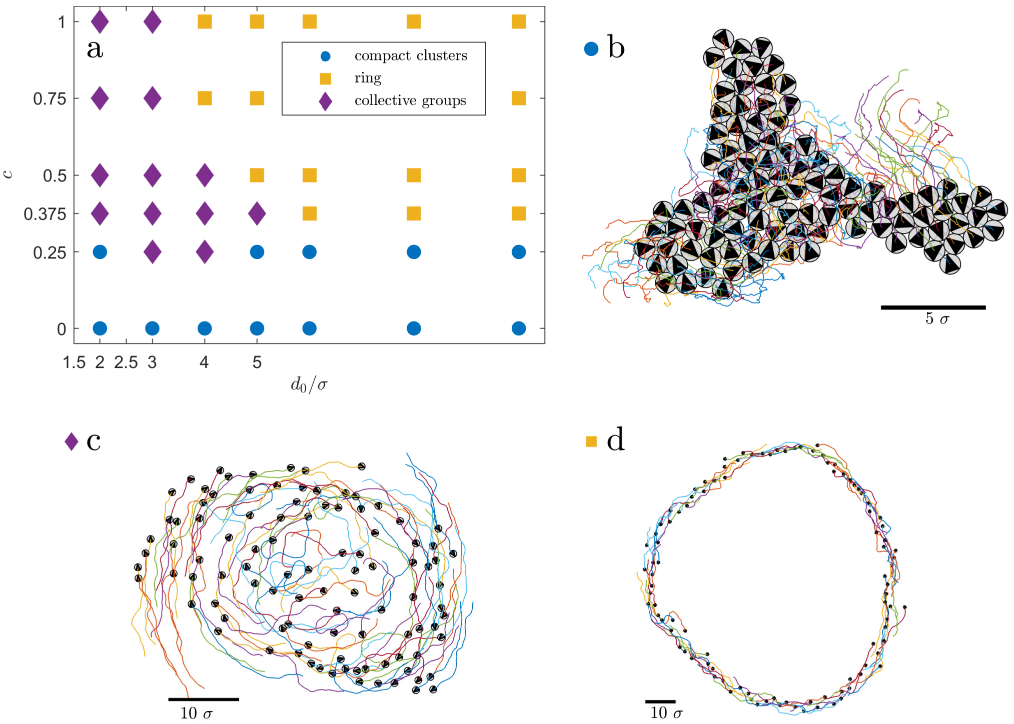

Figure 1: **a** Phase diagram of different group topologies (compact clusters, rings, collective groups) observed collective behavior in the d_0 vs c parameter space. **b-d** Snapshots of the group configurations in the NESS (**b**: cluster, **c**:group, **d**: ring). The WRG and SRG configurations as discussed in the main text correspond to the group phase.

2. Movies

Selected animated trajectories are provided to give a more intuitive insight into the learned collective behavior. Examples are given for the strongly rotating and weakly rotating group cases. Individuals are depicted as isosceles triangles pointing in the direction of orientation. Traces of selected individuals are shown for the last 200 time steps. Additional to the translation in the 2D plane the development of the DOD normalized to the maximal DOD of corresponding individuals is shown for the same time frame. It should be noted that there is considerably less training during the transient from a loose to a dense group compared to in the steady state. As a consequence, the observed spinning motion of individual particles in the beginning of the video "1-SRG-aggregation" might not be the optimal solution. In addition, at low group densities the magnitude of the observables (scaling with the reciprocal next neighbor distance) becomes rather small. Therefore, the particles are seeking to improve their observables by scanning their neighborhood for locally dense regions of neighbors which eventually leads to particle spinning.

1) "1-SRG-aggregation": Aggregation of the strongly rotating group ($c = 1.5\sigma$, $d_0 = 0.375$). Individuals start from a random initial position and coalesces into one collectively rotating group after passing through an intermediate state consisting of smaller rotating aggregates. The movie starts at time step 0 and goes up to time step 1200 with 80 actions per second (aps). Traces are drawn for 200 times steps.

2) "2-SRG-steady_state": Non equilibrium steady state (NESS) of the strongly rotating group ($c = 1.5\sigma$, $d_0 = 0.375$). The full group forms one collectively rotating swirl. The movie covers time steps 2000 to 3000 with 67 aps. Traces are drawn for 200 time steps.

3) "3-WRG-steady_state": NESS of the weakly rotating group ($c = 1.0\sigma$, $d_0 = 0.375$). The strongly cohesive group shows a complex behavior including multiple sub-swirls and travel of individual particle through the while group. The movie covers time steps 2000 to 3000 with 67 aps. Traces are drawn for 200 time steps.
