# Supplementary figures and images for: Dynamics and risk sharing in groups of selfish individuals
Source: J Theor Biol. 2023 Apr 7;562:111433. doi: 10.1016/j.jtbi.2023.111433 (PMC10020420; doi:10.1016/j.jtbi.2023.111433)

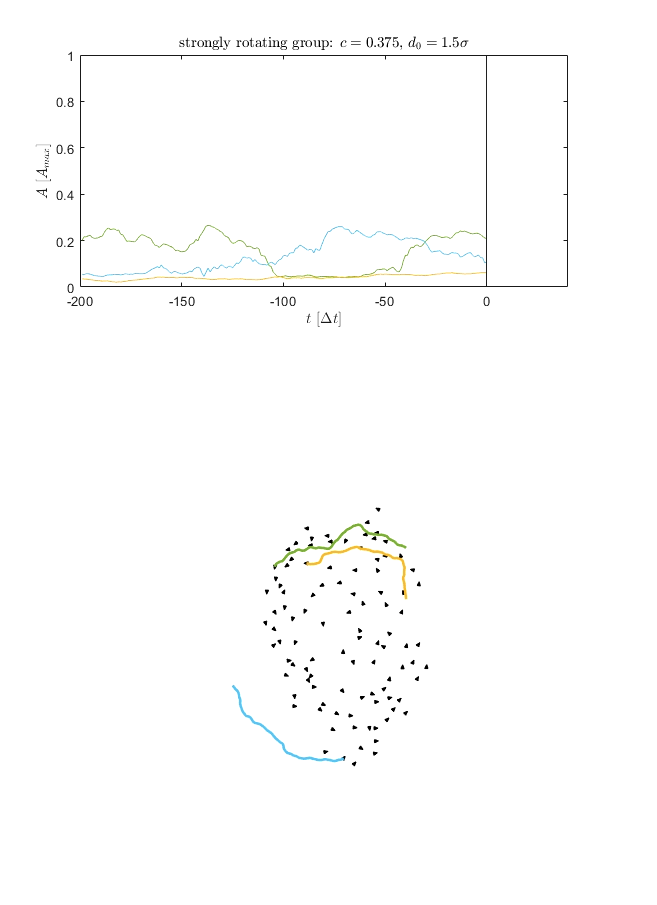

Supplement: MMC S5 — . [file mmc5.zip › still1.PNG]

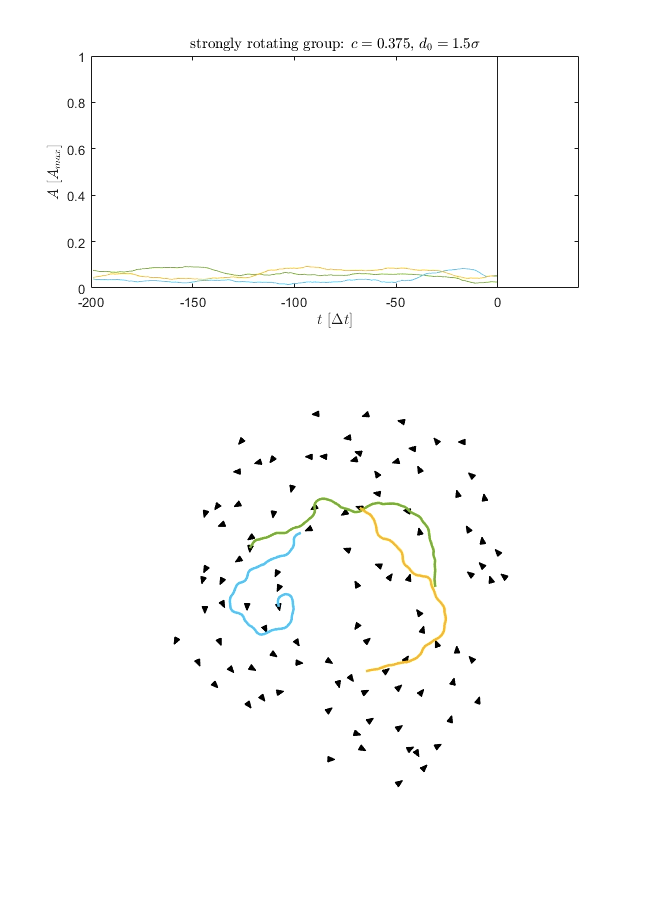

Supplement: MMC S6 — . [file mmc6.zip › still2.PNG]

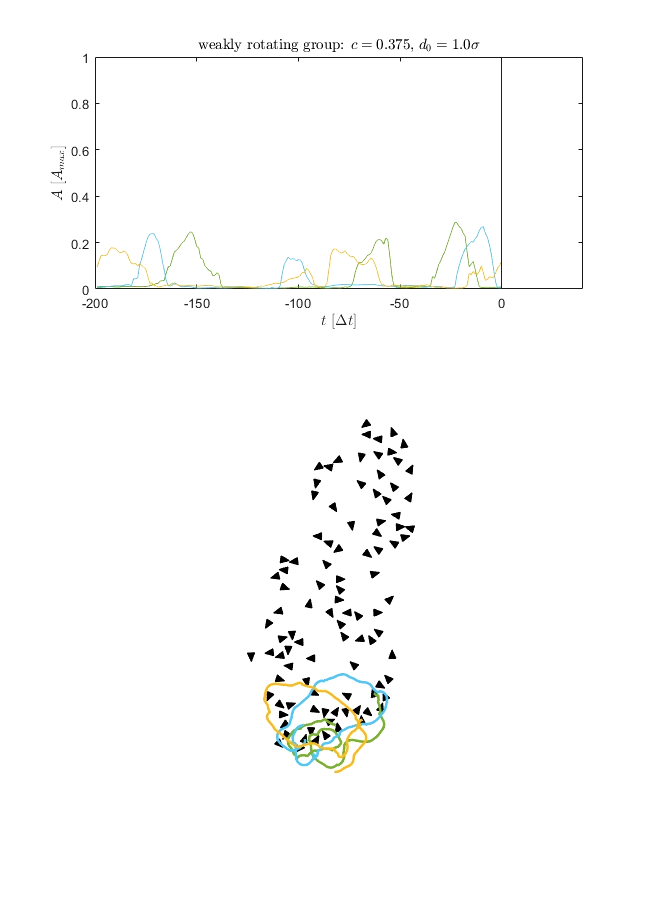

Supplement: MMC S7 — . [file mmc7.zip › still3.PNG]
